# Recent Advances in Microbial-Assisted Remediation of Cadmium-Contaminated Soil

**DOI:** 10.3390/plants12173147

**Published:** 2023-08-31

**Authors:** Usman Zulfiqar, Fasih Ullah Haider, Muhammad Faisal Maqsood, Waqas Mohy-Ud-Din, Muhammad Shabaan, Muhammad Ahmad, Muhammad Kaleem, Muhammad Ishfaq, Zoya Aslam, Babar Shahzad

**Affiliations:** 1 Department of Agronomy, Faculty of Agriculture and Environment, The Islamia University of Bahawalpur, Bahawalpur 63100, Pakistan; usmanzulfiqar2664@gmail.com; 2 Key Laboratory of Vegetation Restoration and Management of Degraded Ecosystems, South China Botanical Garden, Chinese Academy of Sciences, Guangzhou 510650, China; fasihullahhaider281@gmail.com; 3 University of Chinese Academy of Sciences, Beijing 100039, China; 4 Department of Botany, The Islamia University of Bahawalpur, Bahawalpur 63100, Pakistan; faisal.maqsood@iub.edu.pk; 5 Institute of Soil and Environmental Sciences, University of Agriculture, Faisalabad 38040, Pakistan; mohyuddin.waqas5@gmail.com; 6 Department of Soil and Environmental Sciences, Ghazi University, D. G. Khan 32200, Pakistan; 7 Institute of Marine and Environmental Technology, University of Maryland Center for Environmental Science, Baltimore, MD 21202, USA; 8 Land Resources Research Institute (LRRI), National Agricultural Research Centre (NARC), Islamabad, Pakistan; mshabaan@parc.gov.pk; 9 Department of Agronomy, University of Agriculture, Faisalabad 38040, Pakistan; ahmadbajwa516@gmail.com (M.A.); ishfaq2727@gmail.com (M.I.); 10 Department of Botany, University of Agriculture, Faisalabad 38040, Pakistan; kaleemakmal5798@gmail.com; 11 Department of Agriculture, Extension, Azad Jammu & Kashmir, Pakistan; 12 Soil and Environmental Biotechnology Division, National Institute for Biotechnology and Genetic Engineering, Constituent College of Pakistan Institute of Engineering and Applied Sciences, Faisalabad, Pakistan; 13 Tasmanian Institute of Agriculture, University of Tasmania, Hobart, TAS 7001, Australia

**Keywords:** cadmium toxicity, bioremediation, microbes, mechanism, recent advancements

## Abstract

Soil contamination with cadmium (Cd) is a severe concern for the developing world due to its non-biodegradability and significant potential to damage the ecosystem and associated services. Industries such as mining, manufacturing, building, etc., rapidly produce a substantial amount of Cd, posing environmental risks. Cd toxicity in crop plants decreases nutrient and water uptake and translocation, increases oxidative damage, interferes with plant metabolism and inhibits plant morphology and physiology. However, various conventional physicochemical approaches are available to remove Cd from the soil, including chemical reduction, immobilization, stabilization and electro-remediation. Nevertheless, these processes are costly and unfriendly to the environment because they require much energy, skilled labor and hazardous chemicals. In contrasting, contaminated soils can be restored by using bioremediation techniques, which use plants alone and in association with different beneficial microbes as cutting-edge approaches. This review covers the bioremediation of soils contaminated with Cd in various new ways. The bioremediation capability of bacteria and fungi alone and in combination with plants are studied and analyzed. Microbes, including bacteria, fungi and algae, are reported to have a high tolerance for metals, having a 98% bioremediation capability. The internal structure of microorganisms, their cell surface characteristics and the surrounding environmental circumstances are all discussed concerning how microbes detoxify metals. Moreover, issues affecting the effectiveness of bioremediation are explored, along with potential difficulties, solutions and prospects.

## 1. Introduction

In the last two decades, the quality of human life has improved significantly. However, developmental activities have occurred at the expense of the environment’s quality [1]. Soil and the environment are contaminated due to higher concentrations of metalloids and heavy metals (HMs) resulting from rapidly expanding industrial wastes, excessive use of automobiles, resource extraction, petrochemical spillage, metallurgy and anthropogenic activities [2]. A heavy metal is any metallic substance with a relatively higher density and is toxic even at low concentrations [3]. Heavy metals include elements such as aluminum (Al), arsenic (As), antimony (Sb), beryllium (Be), cadmium (Cd), chromium (Cr), copper (Cu), lead (Pb), mercury (Hg) and nickel (Ni) [4]. The persistent nature of these toxic HMs causes harm to humans, plants and animals at higher levels [5].

Cadmium is one of the most dangerous HMs to living organisms [6], mainly due to its higher toxicity and severe extent of bioaccumulation [7]. It adversely impacts human health by accumulating in the kidney and causing renal tubular damage and emphysema [8]. Cd has persisted in soil for decades, depending on multiple factors, including soil type, redox potential, pH, clay contents, organic matter, plant uptake and leaching [9]. Cd presents a unique concern due to its notable mobility in soil environments. Unlike some heavy metals, Cd exhibits a relatively high degree of mobility within soils, facilitated by factors such as soil pH, organic matter content and redox potential. This mobility renders Cd more hazardous even at relatively low soil concentrations, as it can readily leach into groundwater and accumulate in crops, posing environmental and human health risks. Cd toxicity negatively affects plant functioning by inhibiting carbon fixation, reducing chlorophyll synthesis and minimizing photosynthetic activity [10]. Cd-induced phytotoxicity leads to plant morphological alterations, such as chlorosis and the suppression of lateral root formation [11]. Additionally, Cd exposure induces osmotic stress in plants by reducing relative leaf water content, stomatal conductance and transpiration, ultimately leading to tissue damage [12]. Furthermore, the toxicity of Cd results in the overproduction of reactive oxygen species (ROS), damaging plant membranes and destroying cell organelles [13]. Cd toxicity also reduces the uptake and transportation of mineral elements, leading to stunted growth with ultimate yield penalties on field crops [14]. The increased mobility of Cd underscores the urgency of effective remediation strategies to mitigate its potential widespread contamination and its subsequent adverse effects on ecosystems and agriculture.

Cd remediation mitigates or eliminates Cd contamination from environmental systems, particularly soil. Cadmium, a highly toxic heavy metal, poses significant health and ecological risks even at relatively low concentrations due to its mobility within soils [12,15]. This process involves various strategies to reduce Cd’s presence, minimizing its potential harm to human health, ecosystems and agricultural productivity. Remediation methods can be broadly categorized into physicochemical approaches, which involve chemical treatments and physical processes, and bioremediation, which employs living organisms such as microorganisms and plants to transform or remove Cd from the soil matrix. The most commonly employed approaches include chemical oxidation and reduction, precipitation, electrochemical treatment, solvent extraction, ion exchange, filtration, reverse osmosis, recovery by evaporation and soil washing with chelating chemicals [16,17]. However, one major drawback of these traditional processes is the creation of toxic heaps, sludge and secondary pollutants [18]. Therefore, it is necessary to continuously monitor the stability of immobilized HMs such as Cd [19]. Moreover, conventional remediation techniques can only remove Cd to a certain degree. In addition, traditional remediation requires expensive chemicals, significant energy and investment [20].

In contrast to physicochemical procedures, bioremediation is an environment-friendly technique that utilizes plants and microorganisms (such as fungi, bacteria and algae) to aid in the restoration of contaminated soil to its original state [21]. Bioremediation harnesses the natural metabolic capabilities of these organisms to convert Cd into less harmful forms, offering a sustainable and eco-friendly solution to Cd contamination. Biological techniques such as biosorption and bioaccumulation offer an advantage in removing HMs from polluted resources [18,22]. In a natural ecosystem, microbes are widely distributed and thrive in HM-polluted environments [12]. However, the ability of microbes to remediate contaminants can halt when they run out of food [23]. An enrichment method for the isolation of microbes that combines the properties of (1) the degradation of a chosen pollutant and (2) excellent root colonization has been developed [24,25] to ensure that these microbes can access the best available food source in soil, namely root exudates [26]. Plant root exudates, including organic acids, alcohols and sugars, serve as energy sources for soil microflora and promote microbial activity and growth [27]. According to Sabae et al. [28], some root exudates may also function as chemotactic signals for microbes. Furthermore, plant roots enhance water movement and loosen the rhizosphere, which improves microbial colonization [29,30]. As a result, these microbes transform hazardous HMs into non-toxic forms. Throughout bioremediation, these microbes transform organic pollutants into end products, including H_2_O, CO_2_ and metabolites, which are the primary substrates for cell growth [31]. Microbes maintain a defense system against HM contamination in the rhizosphere via two mechanisms: (i) the biosynthesis of enzymes that break down specific contaminants and (ii) persistence that can withstand associated HMs [18].

Despite numerous individual efforts to evaluate the potential of various microbes for remediating Cd, such as bacteria, fungi and algae, no comprehensive review covers the multivariate features of plant growth-promoting microbes and their strategies and mechanisms for decontaminating Cd-contaminated soil. This review covers some new aspects and dimensions of the bioremediation of Cd-contaminated soils. Here, we mainly review the recently published literature from 2010 to 2022. The main objective of this review is to highlight the bioremediation potential of various microorganisms, especially bacteria, fungi and algae, individually and in combination with plants. Different mechanisms, i.e., indirect and direct mechanisms, adapted by microorganisms to detoxify Cd, are also discussed. Furthermore, factors, i.e., water content, temperature, pH, nutrient availability, moisture content and pollutant bioavailability, which can influence the bioremediation of Cd in contaminated soil, are also explored. Finally, the present review explores field application knowledge through case studies, challenges and prospects.

## 2. Review Methodology

The review methodology employed in this study involved a comprehensive search for peer-reviewed research articles using a range of relevant keywords. The keywords used in the search included terms such as “contaminant”, “Cd uptake”, “toxicity”, “accumulation”, “dynamics”, “seed germination”, “oxidative stress”, “antioxidant enzymes”, “photosynthetic rate”, “growth patterns”, “plant physiology”, “lipid peroxidation”, “nutrients uptake”, “mitigation measures”, “microbes”, “immobilization”, “bioremediation”, “mechanism”, “PGPRs”, “membrane and enzyme technology”, “genetic and metabolic engineering”, “metagenomics” and “nanoparticle”.

Six prominent databases were utilized to ensure a wide range of literature sources: Sciencedirect, Google Scholar, Web of Science, Researchgate, Scopus and freefullpdf. These databases are renowned for their vast collections of peer-reviewed articles and are widely recognized in the academic community. The search strategy resulted in identifying approximately 336 relevant articles published between 2010 and 2023. These articles were selected based on their relevance to the research topic and the inclusion of pertinent information regarding the effects of contaminants, specifically Cd, on various aspects of plant biology and physiology and bioremediation techniques to decontaminate Cd. To further enhance the comprehensiveness of the review, the reference lists of the identified articles were examined, and any additional relevant papers cited within these articles were also reviewed. By employing this rigorous methodology, the researchers aimed to ensure that the review encompassed a wide range of up-to-date and credible information on the subject matter, thereby strengthening the overall validity and reliability of the findings presented in the study.

## 3. Sources of Cadmium 

According to an annual United Nations Environment Program (UNEP) report, the amount of Cd released into the environment via different sources varies between 150 and 2600 tons [32]. Natural sources of Cd include rock weathering and air soil particles, e.g., from deserts, sea spray, forest fires, biogenic materials, volcanoes and hydrothermal vents [33]. Various rock types contain significant amounts of Cd, ranging from 0.006 to 8.4 ppm. It is estimated that wind-blown ash deposits approximately 0.25 × 10^6^ kg of Cd annually, volcanic eruptions contribute 0.5 × 10^6^ kg, wildfires contribute 0.01 × 10^6^ kg, and salt seal aerosols contribute 0.002 × 10^6^ kg [34]. Cadmium can enter the soil via the long-term application of fertilizers, pesticides and animal manure [35]. 

## 4. Cadmium Toxicity and Plants

### 4.1. Seed Germination and Seedling Growth

Seed germination is considered the most essential activity following the onset of emergence and is accompanied by a release from dormancy [36]. Growth regulators, such as gibberellic acid (GA), auxin and abscisic acid (ABA), regulate seed germination and early seedling growth [37]. Phytohormones GA and ABA work antagonistically, by which elevated levels of ABA inhibit seed germination and regulate seed dormancy, and GA induces germination [36]. The germination mechanism depends on the seeds’ GA/ABA ratio, which acts as a central hub during integration with environmental stresses [38]. Some other plant hormones, including strigolactones, cytokinin and brassinosteroids, either induce or retard seed germination [39]. Cd inhibits germination and reduces the growth of germinating seedlings [40,41]. The suppression of seed germination mainly occurs due to inhibiting metabolic and physiological processes [42,43]. Cd toxicity in seeds reduces water absorption capacity and starch digestion and impairs the growth of growing embryos [44]. Besides hormonal disturbance, ROS that are too low cause the failure of normal seed germination, and excessive Cd toxicity leads to higher ROS (H_2_O_2_, free radicals, singlet O_2_) and damages the growing embryo [45]. Excess ROS accumulation occurs due to low levels of cellular antioxidants (SOD, POD and CAT) [46]. At low levels, H_2_O_2_ favors seed germination and acts as an oxidative spell of germination. A low concentration of H_2_O_2_ actively oxidizes several proteins, enzymes and mRNAs [47]. Furthermore, Cd contamination impairs germination by reducing seed water uptake, blocking the transport of soluble sugar to the embryonic axis and decreasing the starch release capacity of the embryo due to the inactivation of α-amylase [48]. The inactivation of α-amylase mainly occurs when chemically similar Cd ions starve beneficial Ca ions in seeds [36]. During the early phase of seed germination, Cd and Ca-calmodulin compete to replace Cd with Ca ions for regulating normal germination and maintaining membrane integrity [43].

### 4.2. Cadmium-Induced Changes in Growth and Development

**Figure 1:**
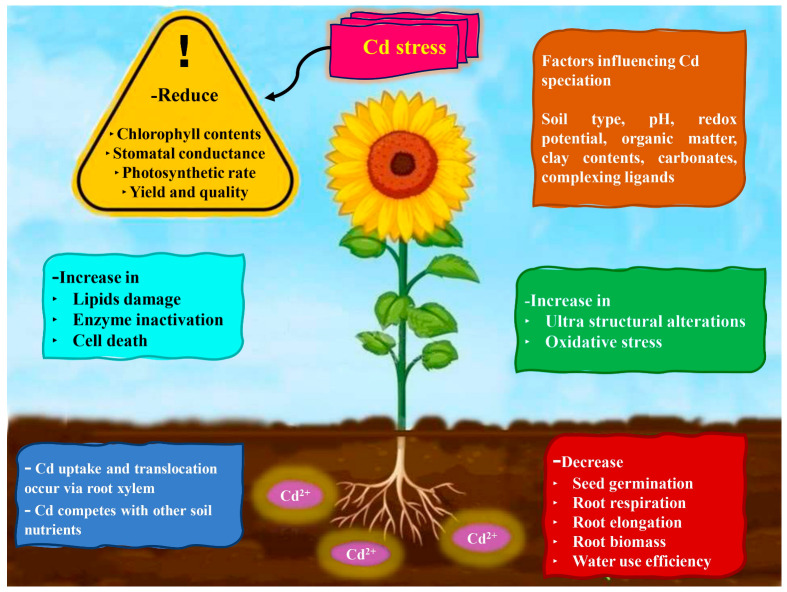
Factors affecting cadmium speciation in soil, and its toxic impacts on plant physiology, morphology and metabolism.

Cd is considered a non-essential element for normal plant growth and development. It can damage growth-related traits in various plants [49], as shown in Figure 1. Cd ions bind to functional proteins and make them dysfunctional. This leads to the degradation of the photosynthetic apparatus and a reduction in photosynthetic pigments, ultimately reducing biomass production and plant growth [50]. The absorption and translocation of Cd^2+^ reduce the leaf surface area and, subsequently, photosynthetic products [49]. Long-term exposure to Cd at the root level leads to mucilaginous, decomposing and necrotic roots. These changes ultimately lead to leaf chlorosis, rolling and premature leaf falls [15,51]. Additionally, excessive Cd accumulation in the rhizosphere disturbs the root system, inhibiting primary and lateral root growth, causing root stiffness and turning the roots brownish and twisted [52]. Cd toxicity also reduces mitotic divisions and proliferates the cortical cells of roots, thereby reducing root length and minimizing dry biomass [53]. To mitigate the adverse effect of Cd on roots, plants increase root parenchyma and cortical cell area to make an efficient flow of nutrients and water [54].

### 4.3. Impact on Amino Acids, Proteins and Organic Osmolytes

During HM stress, plants synthesize various low-molecular-weight organic osmolytes, such as amino acids, total soluble sugars and proteins [55]. These organic osmolytes act as signaling molecules and free radical scavengers and can modulate the stomatal aperture while reducing oxidative stress [56]. Amino acids regulate pH, enzyme synthesis and redox homeostasis [57]. During HM stress, amino acids upregulate osmotic adjustments, maintain integral proteins and ion homeostasis, neutralize redox potential and scavenge ROS by maintaining plant antioxidant levels [58]. However, Cd toxicity induces damage to proteins in the cell cytoplasm [49]. Its uptake by roots can reduce proteins via increased H_2_O_2_, LPX and free radicals [59].

### 4.4. Plant Water Relations

Exposure to Cd stress leads to adverse changes in the water status of plants [60]. Cd reduces the extent of water availability and nutrient translocation at the root level, disturbing short-distance symplast and apoplast pathways [53]. Excess Cd ions in root cells also lower the water status above ground [61]. Water balance disturbances can lead to low membrane integrity during lipid peroxidation [62]. Cd negatively influences the physiological mechanism of the cell water status and gas exchange, impairing plant metabolic processes [63]. Exposure to Cd in plants results in imbalanced nutrient and water uptake, reducing photosynthetic performance and biomass production [64]. Moreover, low water availability increases Cd ion sequestration in root cells and causes oxidative damage to root cells [65].

### 4.5. Impact on Photosynthesis

Cadmium poses a severe threat to the photosynthetic system of plants [66], and its accumulation in leaves can cause oxidative stress and a decline in transpiration rate, leading to stomatal closure [67]. Exposure to Cd damages various crucial components of plants’ photosynthetic systems, including photosystems, reaction centers and antenna complexes [68]. Photosynthetic efficiency in leaves depends on the availability of Fe^2+^ and the synthesis of other accessory pigments. Cd can inhibit the activity of Fe^3+^ reductase, causing a reduction in Fe^2+^ and leading to a decline in the photosynthetic performance of plants [69]. Cd-induced low pigment synthesis can result in a minimum density of chloroplast and chlorosis [70]. Furthermore, Cd can disrupt the shape of chloroplast and inflate thylakoids [71]. The toxicity of Cd also adversely impacts mesophyll structure and poorly developed anatomical structures that alter the biochemistry of photosynthesis. Negative interactions with SH groups can inhibit photosynthetic enzymes [72]. The impact of Cd toxicity in different plant species is presented in Table 1.

## 5. The Role of Microbes in the Bioremediation of Cd-Contaminated Soils 

Microorganisms have been utilized for the remediation of HM pollution through various techniques, including the immobilization, adhesion, processing, oxidation and volatilization of HMs [12,15]. To maximize bioremediation efficiency, it is imperative to identify the underlying mechanisms that govern the behavior and proliferation of microorganisms in contaminated sites and their reactions to environmental fluctuations [86]. Bioremediation techniques include interactions between microbes and metals, biosorption, biotransformation, biomineralization, bioaccumulation and bioleaching. Microorganisms that depend on chemicals for growth and development can remove them from soil [87]. In addition to dissolving metals, microbes can oxidize and reduce transition metals. Cell membranes can be harmed by different organic solvents [31]. However, microbial cells can evolve defense mechanisms, such as creating solvent efflux or hydrophobic pumps, to prevent damage to the outer membrane [88]. Plasmid-encoded or energy-dependent metal efflux systems have been found in numerous bacteria that resist metals such as Cr, Cd and As [89]. The microbe-assisted phytoremediation of Cd from soil under different experimental settings is detailed in Table 2.

### 5.1. Remediation of Cd by Bacteria

Bacterial biomasses, both dead and alive, can be used in bioremediation. Cd can be removed through the processes of biosorption and bioaccumulation [111]. By modifying the cell wall–plasma membrane complex and depositing Cd into the cell wall, bacteria can resist the harmful effects of Cd [15]. Cd enters bacterial cells through the absorption mechanisms of divalent cations such as manganese (Mn^2+^) or zinc (Zn^2+^) [112]. The surface of bacterial cells contains several functional groups, including carboxyl, phosphonate, sulfonate, hydroxyl and amide groups, which can absorb Cd from soil solutions [113]. Some of the most effective Cd-bioremediating bacteria include *Streptomyces* R25, *Fomitopsis pinicola* CCBAS 535 [114] and *Pseudomonas aeruginosa* [111]. Compared to the control, *Bacillus subtilis* L. and *Saccharomyces cerevisiae* L. absorbed 75.76 and 69.56% of Cd from contaminated soil after five days of inoculation [26]. *Bacillus subtilis* L. can improve water absorption and minimize electrolyte leakage (EL) to promote plant growth and reduce Cd toxicity [115]. *Bacillus licheniformis* L. increases the dispersion of Cd and its accumulation in plants under contaminated soil conditions, which lowers the amount of hazardous Cd in soil [25,116]. In wheat (*Triticum aestivum*), the inoculation of *Bacillus siamensis* L. reduced Cd toxicity by reducing the malondialdehyde (MDA) content and increasing the catalase (CAT) and superoxidase (SOD) contents [117]. Moreover, the inoculation of *Bacillus siamensis* L. increased wheat crop yield under Cd stress by increasing membrane stability, total soluble sugars, amino acid synthesis and photosynthetic activity [117].

Moreover, the application of plant growth-promoting rhizobacteria (PGPR) can be a significant factor in bacterial-assisted Cd bioremediation [12,15]. PGPR inoculants, such as *Rhodococcus* sp. 4N-4, *Flavobacterium* sp. 5P-4, *Variovorax paradoxus* 2C-1, [118], *Flavobacterium* sp., *Kluyvera ascorbata* SUD165 and SUD165/26, *Pseudomonas tolaasii* RP23, *P. Fluorescens* RS9, *Rhodococcus* sp., *Variovorax paradoxus* [118,119], *Pseudomonas aeruginosa* [120,121], *Pseudomonas* sp., *Bradyrhizobium* sp., *Ochrobactrum cytisi* [122], *Bacillus megaterium* [123] and *Rhodobacter sphaeroides* [18], have significantly been used to mitigate Cd toxicity in various agricultural and horticultural crops grown in Cd-contaminated soils. In addition, PGPR release antifungal chemicals, such as hydrogen cyanide, and mobilize nutrients, particularly phosphates, from soil to defend plants from fungal disease [124]. Certain PGPR release 1-aminocyclopropane-1-carboxylate (ACC) deaminase, which helps plants recover from biotic and abiotic stress [124,125]. Therefore, PGPR can increase species’ capacity to remove Cd through bioremediation and may be used in phytoremediation techniques.

### 5.2. Remediation of Cd by Fungi

Mycoremediation is the process of using fungi to bioremediate Cd [18]. It involves utilizing fungi’s extracellular enzymes or potential metabolic capacity to reduce organic and inorganic contaminants in natural resources [126]. Fungi have been widely accepted for their involvement in the remediation of Cd due to their physical contact, low cost, wide availability, increased cell-to-surface ratio and fungal enzymatic activities with the surrounding environment, as well as their ability to be farmed on a large scale [127]. Fungi have demonstrated enormous physiological and metabolic capacity to digest harmful substances and lower the associated environmental concerns with these molecules via chemical changes or affecting chemical bioavailability [128]. Mycoremediation involves intra- and extracellular precipitation, valence transformation and an active uptake mechanism [129,130]. Mycelium’s role in fungus degradation makes it an active participant in bioremediation. Fungal mycelia penetrate the air spaces of polluted soils [131]. They secrete extracellular enzymes, organic acids and complex organic compounds, which aid in the solubilization and chelation of metal ions [132].

Mycoremediation involves several mechanical routes, including extrusion, sequestration, biotransformation, avoidance/exclusion and biodegradation [130]. It is a useful technology regarding the hyperaccumulation of contaminants. Hyper-accumulators tend to accumulate pollutants with low concentrations due to their low biomass, whereas fungi accumulate more contaminants [133]. Interactions between fungal species and hyperaccumulator plants, leguminous plants and other herbs can lead to an efficient phytoremediation strategy [134]. Arbuscular mycorrhizal fungus (AMF) creates a physical connection directly between soil and plants, increasing the rhizosphere surface area and improving nutrient absorption [135]. However, the increased surface area also increases the vulnerability of plants to contaminants. Several regulating factors affect exposure and, subsequently, metal toxicity, such as plant and fungal species, their ecotypes, the bioavailability of pollutants, soil properties, soil fertility, root growth and light intensity [136]. In Cd bioremediation, *Microsphaeropsis* sp. LSE10 was found to have the highest removal efficiency of 247.5 mg g^−1^ compared to other fungal species [137]. Mycorrhizae can act as a barrier that prevents contaminants from passing through to plants. Metals can also attach to fungal hyphae, indicating that they may react to contamination [138,139]. Fungal vesicles, spores, extraradical mycelia and intraradical mycelia are essential for the accumulation of metals and the chelation of contaminants [140].

Moreover, plants inoculated with AMF may produce molecules that chelate Cd complexes, such as phytochelatins, metallothioneins and glutathione [141]. According to Zhang et al. [139], glomalin produced by AMF mycelia can also bind more metals. Hence, it can significantly immobilize HMs and promote host plant tolerance to harsh situations. Certain fungal species, such as *Trichoderma* spp. and *Piriformospora indica*, are adaptable due to their ability to grow in soils with high contaminants [142] Data suggest that Trichoderma metal tolerance strains can significantly influence the bioaccumulation of Cd and other contaminants [143]. *Trichoderma simmonsii* L. (UTFC 10063) has the potential to bioaccumulate Cd and reduce its Cd toxicity by 46.1% [144]. *Aspergillus niger* L. significantly eliminated Cd ions in soils by 84% [145]. Reports indicate that *Trichoderma atroviride* L. affects rapeseed’s uptake and the translocation of Cd, Ni and Zn [146]. Other fungal varieties, such as *Trichoderma mutant* L. [147], *Talamyces emersonii* L., Basidiomycetes [148,149], *Trichoderma harzianum* L., *Trichoderma tomentosum* L. and *Trichoderma asperellum* L. [150], aid in the remediation of Cd from contaminated agricultural soils [144]. It is important to exploit fungi that can remediate contaminated soil through bioaccumulation, bio-volatilization and biosorption to reduce Cd contamination from agricultural soils.

### 5.3. Remediation of Cd by Algae

Phyco-remediation involves using algae and cyanobacteria for Cd removal, assimilation, degradation, etc. [151]. Due to its greater algal availability, low operational costs, low sludge generation, facile application and low nutritional demand, bioremediation with algae is advantageous [152]. In controlled circumstances, Kumar et al. [153] investigated the brown color variant of *Kappaphycus alvarezii* that absorbed 3.365 mg of Cd 100 g^−1^ fresh weights. Previous studies have shown that the Chlorella, Ulva, Sargassum, Fucus and Ascophyllum species can absorb HMs. Cd accumulates on several cell wall layers of *Spirulina maxima* [154,155,156]. Of Microcystis aeruginosa, 90% have a strong affinity for Cd^2+^. The Cd^2+^ concentration of 1 mg L^−1^ showed strong population growth for *Porphyridium cruentum* and reduced its population growth at 5 mg L^−1^ [157]. According to Saunders et al. [158], Cd can be removed from coal-fired power plant wastewater using the algae species *Hydrodictyon*, *Oedogonium* and *Rhizoclonium*. *Spirogyra hyaline*’s dry algal biomass has been shown by Kumar and Oommen [159] as a useful biosorbent for Cd remediation. The effectiveness of *Chlorella vulgaris* and *Chlamydomonas reinhardtii* in removing Cd was established by Kotrba et al. [160]. According to Tuzen and Sari [161], *Chlamydomonas reinhardtii* biomass can be biosorbents in the removal of Cd^2+^. The algal species or strains, sorption process, immobilization techniques, manipulation of Cd binding sites, economic viability of remediation technologies, etc., all play a role in the phyco-remediation of Cd commercially. Table 3 thoroughly summarizes the efficiency of various bacterial, fungal and algal cultures for Cd bio-removal.

## 6. Mechanisms Involved in Bioremediation by Microbes 

### 6.1. Direct Mechanisms

#### 6.1.1. Nitrogen Fixation

Nitrogen (N) is a vital nutrient for plant growth, as it is required for chlorophyll production, photosynthesis and cell division. N is often the key limiting element for plant growth and development [196]. It is the most abundant element in Earth’s atmosphere but only exists in its inert state in certain modified prokaryotes, such as some cyanobacteria, actinomycetes and eubacteria [197]. Legumes can form symbiotic relationships with rhizobia that fix N in soil, such as *Azorhizobium*, *Allorhizobium*, *Bradyrhizobium*, *Mesorhizobium*, *Rhizobium* or *Sinorhizobium* [198]. Several PGPR, such as *Rhizobium*, which forms a symbiotic relationship with root nodules of leguminous plants, can continually transform atmospheric N into nitrate and ammonium for plant use by producing nitrogenase enzymes [199].

Certain PGPR may improve plant absorption of N in rhizosphere soil [200]. For instance, inoculation with PGPR can boost N absorption and encourage the development of tomato (*Solanum lycopersicum*) plants under a rising N supply [201], similar to Singh et al. [202], who selected 22 isolates from the rhizosphere of sugarcane (*Saccharum officinarum*). Similarly, several *Bacillus* isolates have been shown to exhibit N-fixing and biocontrol abilities. Rhizobia produce Nod Factors (NFs) in response to plant root exudates that include (iso) flavonoids, which start the symbiotic process leading to the initiation of bacterial infection. Nodules, which develop in roots and, in rare instances, stems, result from this molecular conversation. Plants stipulate a carbon supply to microbes to fuel the symbiotic biological N fixation (BNF) process and a microaerophilic environment within nodules, consistent with nitrogenase (Nase) complex functioning. The Nase enzyme converts dinitrogen from the atmosphere to ammonia, which is subsequently converted into organic forms and is expelled from nodules to support plant development. Because N-limited conditions are a typical characteristic of metal-contaminated soils, symbiotic BNF also makes legumes the ideal pioneers to invade and repair the quality and health of these habitats [203]. This ability, combined with the deep-dwelling root systems and large biomass of legumes, makes them suitable candidates for the effective phytoremediation of Cd [204].

#### 6.1.2. Phosphate Solubilization

Phosphate-solubilizing bacteria (PSB) have been found to enhance plant growth in Cd-contaminated soils by providing phosphorus (P), making them useful for the remediation of Cd. Despite being a crucial component for plant development, soil seldom contains enough P. Both organic (Po, average 50%) and inorganic (Pi, average 50%) forms of P are found in nature [205,206]. Roots can absorb them because none are soluble (typically no more than 5%) [207]. Plants may absorb monobasic (H_2_PO_4_) and dibasic (HPO_4_^2−^) ions. Pi is mostly soluble when the soil’s pH decreases due to the synthesis of low-molecular-weight organic acids. On the other hand, phosphoric esters are hydrolyzed by phosphatase during the mineralization of organic P [208]. PSB activity produces enough P, significantly reducing the need for chemical fertilizers [209]. PSB dissipates inorganic phosphates through organic acid secretion, which increases phosphate solubility by ionizing protons, lowering the pH and combining PO_4_^3−^ to create HPO_4_^2−^ or H_2_PO^4−^. The organic acid may also form Al^3+^, Ca^2+^ and Fe^3+^ complexes, making PO_4_^3−^ available for plant uptake. Similarly, PSB-supported phytoextraction may enhance the mobility of Cd in soil, as revealed by various research. Endophytic *Rahnella* sp. JN6 efficiently solubilized 8.8 mg L^−1^ Cd and 133.54 mg L^−1^ phosphate in a liquid culture and increased Cd accumulation in mustard (*Brassica napus*) [210]. *Burkholderia* sp. J62 boosted maize and tomato biomass by solubilizing 25.8 mg L^−1^ Cd and 234 mg L^−1^ phosphate in a culture solution. The favorable impact on plants encourages increased phytoextraction or phytostabilization effectiveness in Cd-contaminated soil [211].

#### 6.1.3. Phytohormone Production

Metals may also affect plant growth and development. Numerous studies have shown that plants grown under metal stress conditions experience damage to their membrane system, which affects the structure and function of organelles and various physiological and biochemical processes in their tissues. Lignans, or phytohormones, are active chemical compounds produced by plants that may have specific physiological effects on plants even in very low quantities [212]. They are strongly connected to root growth and may control various plant life cycle functions. Environmental challenges, such as HM toxicity, harsh temperatures, nutrient deficiencies and drought, which may cause a range of unfavorable physiological and chemical responses in plants, continually affect plant development during phytoremediation [213,214]. PGPR may increase plant tolerance to such stresses and promote plant development in Cd-contaminated soil by preserving the nutritional status and modifying phytohormonal balance by synthesizing plant growth regulators. Several studies have shown that PGPR are important in phytohormone synthesis, which may govern plant growth, development and physiological processes and influence biological and non-biological stress responses. Various PGPR have been identified that produce phytohormones, such as IAA, auxins, cytokinin and gibberellins, during harsh conditions, ultimately promoting plant development and enhancing the plant’s ability to withstand under environmental stress due to Cd contamination [215,216]. The *Bacillus* sp. MT7 strain was isolated from maize rhizosphere soil and tested in a tomato rhizosphere [217]. The results showed that MT7 produced 14.44 g mL^−1^ IAA 4 days after inoculation, promoting plant growth and showing tolerance against Cd stress. *Pseudomonas fluorescens* may boost the wedge’s development and physiological processes (*Sedum alfredii*) by generating IAA and improving plant Cd absorption via modulating Cd expression and transport genes. Pan et al. [218] showed the ability of ABA-producing *B. subtilis* to reduce Cd accumulation in *Arabidopsis thaliana*. Numerous bacterial species can produce gibberellic acid to reduce metal toxicity by lowering Cd absorption and lipid peroxidation, affecting hormonal balance and controlling the activities of proteases, catalase and peroxidase.

#### 6.1.4. Antagonistic Role of PGPR

**Figure 2:**
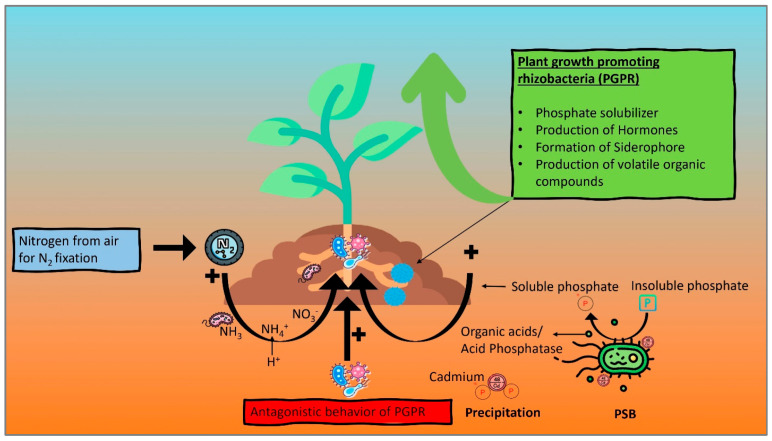
Microbially mediated direct mechanisms for contaminant detoxification.

Antagonizing bacteria are crucial biocontrol agents in the rhizosphere zone, as they protect the plant from disease caused by pathogenic bacteria [219], as shown in Figure 2. Possible mechanisms for antagonistic activity may consist of antibiotics that inhibit pathogenic activity, the place for colonization, competition for nutrients, parasitism and mycophagy [220,221]. Biological control agents are more sustainable options for farming and are well accepted in many nations. Numerous biocontrol agents, including bacteria, fungi and actinomycetes, have been investigated for their potential effectiveness against various phytopathogens. Many have also been found to act on PGPR [222]. Different bacterial species, such as *Pseudomonas*, *Bacillus*, *Klebsiella*, *Azospirillum* spp., etc., have adapted to rhizosphere soil and have effectively defended plants against diseases [216,223]. *Aspergillus flavus*, often found in soil, has the potential to infect crops, and its aflatoxin negatively impacts the majority of crops [224]. A filamentous fungus called *Alternaria alternata* is responsible for causing diseases such as leafspot in Aloe vera and stem canker in tomato plants [225]. The fungus *Fusarium oxysporum* is a typical member of rhizosphere microbial communities, and in most cases, they are non-pathogenic. Pathogenic Fusarium, on the other hand, may attack plant roots and result in Fusarium wilt [226]. Various research has stated that certain soil bacteria, particularly PGPR, may be able to synthesize the cytokinins and gibberellins that control plant growth and development [227]. Using more efficient and genetically engineered bacteria (GEB) has shown a more significant requirement for removing Cd from polluted sites. Metal-binding peptides such as phytochelatins and metallothioneins have been found to improve HM binding. Phytochelatins are known to bind HMs, particularly Cd, with great affinity, by creating thiolate complexes. Phytochelatin coding genes have recently been cloned from plants and fungi and functionally expressed in *Escherichia coli*.

According to Paul and Bhakta [228], GEB, with both a high bioaccumulation capability and a strong affinity for the target metal, have demonstrated that they preferentially collect metal ions from multi-component pollution. The unique Cd transport mechanism and the metallothionein protein significantly enhance GEB’s ability to accumulate Cd^2+^ from multi-component metal-contaminated sites [229]. Dixit et al. [88] discovered that *Mesorhizobium huakuii* modified with an *Arabidopsis thaliana* gene coding for phytochelatins accumulated more Cd^2+^. In addition, according to Hou et al. [230], the amount of Cd accumulation in the recombinant *Escherichia coli* strain was about 25-fold greater than that in the control strain. Oliva-Arancibia et al. [231] found that *Pseudomonas putida* 06909 reduced Cd cellular toxicity. Recently, recombinant *Caulobacter crescentus* strain JS4022/p723-6H, expressing the RsaA-6His fusion protein, could remove 94.3–99.9% of Cd (II), whereas the control strain could only remove 11.4–37.0% [232]. Without calling the findings into doubt, there is significant promise for using recombinant technology to remove target HMs efficiently.

#### 6.1.5. Siderophore Secretion

PGPR secrete a variety of Fe carriers known as siderophores to sustain metabolic activity. These siderophores may make complexes with HMs in the soil to increase their bioavailability via biochemical reactions to facilitate absorption through plants’ roots [216,233]. Low-molecular-weight compounds known as siderophores may bind ferric ions and make them available for microbial cells. PGPR bacteria can synthesize siderophores, which thrive and produce at their best in harsh environmental circumstances with low nutrition availability or under HM toxicity. For instance, Gazitúa et al. [234] identified six bacteria in the rhizomes of plants for culturing in the substrate under harsh conditions, which were isolated from the plant rhizospheres growing in floating tailings. The results demonstrated that bacterial strains improved the aboveground and belowground biomass of plants both by PGPR and metal-resistant bacterial strains. This is primarily because they may produce iron carriers and improve phosphate breakdown, which can lessen the detrimental effects of excessive HM concentrations and a deficiency of heavy elements [235]. Cadmium can also be transported through Fe transporters. However, two different systems are reported to uptake Fe from the soil. The first involves reduced Fe (II) uptake, whereas the second involves chelated Fe (III) uptake. Fe (III)-phytosiderophore complexes (Fe(III)-PS) assist transportation in barley and maize through yellow stripe 1/yellow stripe-like 1 (YS1/YSL1). It has been reported that the SnYSL3 and OsYSL2 proteins transport Cd in black nightshade (*Solanum nigrum*) and *Oryza sativa*, respectively [236].

Many plant-related bacteria make siderophores an essential PGPR feature in delivering iron to plants while protecting them against fungal infections. Cd^2+^ induces the synthesis of green-pigmented ‘pyoverdine’ siderophores in *Pseudomonas*. Mustard and pumpkin plants inoculated with siderophore synthesizing *P. aeruginosa* showed greater iron content in leaves in Cd-contaminated soil. Desferrioxamine E, Desferrioxamine B and coelichelin were identified as Cd-induced siderophores [237]. *Streptomyces tendae* synthesize these siderophores, aiding plant development, enabling soil metal solubilization and increasing Cd and Fe absorption in sunflowers (*Helianthus annus* L.). Siderophore production was demonstrated to boost iron absorption while decreasing Cd uptake in *Streptomyces* [238]. *Pseudomonas putida* enhanced plant growth by producing pyoverdines and reducing Pb and Cd absorption in mung beans. Plants inoculated with siderophore-producing bacteria were shown to either boost or limit Cd absorption, depending on the plant, bacterium and metal combination.

#### 6.1.6. Volatile Organic Compounds

Low-molecular-weight molecules known as volatile organic compounds (VOCs) typically have less than 12 carbon atoms and a vapor pressure of at least 0.01 kPa at an ambient temperature. They may be linked to additional elements, including oxygen (O), nitrogen (N), bromine (Br), sulfur (S), fluorine (Fl) and chlorine (Cl) [239]. These substances are known as biogenic VOCs when living things synthesize them. Research has revealed that these chemicals are significant in various processes supporting plant growth, the induction of systemic resistance (ISR) and plant chemical signaling [240]. Bacterial VOCs such as acetoin and 2,3-butanediol may interact with plants, triggering plant defense and growth promotion mechanisms and assisting host plants in absorbing nutrients such as iron (Fe) and S. VOCs generated by *Bacillus* sp. B55 significantly enhanced the S feeding of *Nicotiana attenuata*. VOCs are important in most PGPR as bioprotectants through induced biopesticides, systemic resistance and phytostimulators. Such actions may help plants develop quicker, which is essential for the successful phytoextraction of Cd-contaminated soil. The volatile dimethylhexadecylamine (DMHDA), which is involved in promoting the growth and development of barrel clover (*Medicago truncatula*) seedlings, is one of these VOCs with advantageous activities in plants and is produced by the rhizobacterium *Artrobacter agilis* strain UMCV2, particularly under Fe deficit conditions [241]. DMHDA boosted the biomass, ferric reductase activity and chlorophyll content.

Additionally, DMHDA induces *M. truncatula* plant roots to release protons that aid in acidifying the rhizosphere, enabling Fe absorption under limited circumstances and increasing the amount of this element in plants inoculated with bacterium [242]. Later research revealed that the UMCV2 strain might live as an endophyte and colonize them in plant tissues. Additionally, there is evidence that VOCs may benefit the rhizosphere and boost plant immune systems. For instance, *Bacillus subtilis* synthesized volatile 2,3-butanediol, stimulating plant growth and physiological reactions. Additionally, 2,3-butanediol-exposed plant roots reacted by producing more root exudates. These findings imply that 2,3-butanediol induces the release of root exudates that regulate the activity of fungi and bacteria in the rhizosphere [243]. Rojas-Solis et al. [244] stated that two *Bacillus* strains synthesized volatile chemicals with synergistic actions to improve plant growth and reduce possible diseases such as *Botrytis cinerea*. Dimethyl disulfide (DMDS), a flammable substance, inhibited the growth of *B. cinerea’s* mycelium. Compounds such as ‘DMDS’ have yet to be assessed for their role in rhizosphere colonization. According to Sharma [245], *Juncus maritimus* synthesizes malonate and oxalate, which function as Cd-complexing agents, boosting Cd transport and solubility in soils. *Pseudomonas* sp. produces organic acids and enhances soil mineral nutrition and Cd availability by increasing host-mediated VOC secretion, dramatically increasing the shoot plant biomass of *Solanum nigrum* and Cd acquisition in aerial portions [245].

### 6.2. Indirect Mechanisms

#### 6.2.1. Production of Antibiotics

To combat damage caused by phytopathogens, the primary approach of plant growth-promoting bacteria is the production of antibiotics. The biocontrol capabilities of bacterial strains such as *Pseudomonas* are mainly dependent on root colonization, the stimulation of plant systemic resistance and the production of antifungal antibiotics [246]. The synthesis of one or more antibiotics is often linked to the potential of rhizobacteria as biocontrol alternatives against plant pathogens. Cd resistance genes are primarily located on plasmids in bacteria, which allow them to resist Cd stress competitively. According to some research, Cd resistance on R plasmids relates to multiple antibiotic resistance [247]. The R plasmid is often found in clinically isolated human infections, such as *Pseudomonas aeruginosa*, *Klebsiella pneumoniae*, *Staphylococcus aureus*, etc. Cd resistance gene loci may be found on plasmids or on chromosomes [248]. The idea of antibiosis, or biocontrol based on the synthesis of chemicals that destroy or impede the development of target pathogens, has been characterized over the last 20 years. Antibiotics are a broad category of organic, low-molecular-weight substances that stop bacteria and other microbes from growing or functioning metabolically. According to Kenawy et al. [249], six groups of antibiotic substances, such as phloroglucinols, phenazines, pyrrolnitrin, cyclic lipopeptides, pyoluteorin and HCN, are most effectively linked to the biocontrol of root diseases.

All these substances are diffusible except for HCN, which is volatile. Lipopeptide biosurfactants synthesized by *Bacillus* and *Pseudomonas* strains have recently been utilized as a biocontrol agent because of their probable positive impact on competitive interactions with fungi, protozoa, oomycetes, bacteria, nematodes and plants. *Pseudomonas* synthesize antibiotics, i.e., HCN, 2,4-diacetyl phloroglucinol (DAPG), amphisin, oomycin A, phenazine, tropolone, tensin, pyrrolnitrin, pyoluteorin and cyclic lipopeptides, and *Bacillus*, *Streptomyces* and *Stenotrophomonas* spp. produce xanthobaccin, kanosamine, oligomycin A and zwittermicin, which have been recognized as antibiotics having antifungal, antiviral, phytotoxic, cytotoxic, antitumor, and antioxidant properties. It has been shown that the antibiotic ‘pyrrolnitrin’, produced by the *P. fluorescens* BL915 strain, defends cotton plants against *Rhizoctonia solani* in Cd-contaminated soil [250]. There have been several studies into efflux processes. For the extrusion of biocides, antibiotics, toxic metals and toxic substances from inside the bacterium into the environment, efflux pumps are composed of integrated membrane proteins. More than 20 potential efflux pumps proteins, either encoded by plasmids or chromosomes, have been described in *S. aureus* up to this point due to developments in genome analysis and bioinformatics [248]. Cd resistance has also been observed in drug-resistant efflux pumps [251]. 

#### 6.2.2. Production of Exopolysaccharides

Since the discovery of the exopolysaccharide (EPS) adsorption potential by bacteria, several studies have already been published on a distinct range of microbial strains and EPSs with the required potential, i.e., the remediation of metal-contaminated soils [252]. Because EPS has a polysaccharide backbone, it is possible to structurally alter it by changing the polymeric length or adding a variety of side chains, non-carbohydrate substituents, functional units, linkages and other bonds in a combinatorial manner. The kind and proportion of the carbon source; abiotic stress elements, including temperature, pH and HMs; and the growth phase of the rhizobacterium, during which synthesis takes place, are all variables that affect the composition of the polysaccharide. The utilization of negatively charged EPSs (EPSs with large anionic functional groups), a feasible biosorbent, must be highlighted in strategies for the remediation of metals using rhizobacterial EPSs [253]. The polymer has a general negative charge due to the presence of numerous ionizable and active non-carbohydrate side chains and functional groups, including structural polysaccharides (fungi); acetamido (chitin group), amine, sulfhydryl and carboxyl groups in proteins; and hydroxyl and phosphate groups and phosphodiester in polysaccharides [254]. Extracellular heteropolysaccharides are polyanionic, unlike homopolysaccharides, because some functional groups interact with polysaccharide backbones [255]. Complexation, ion exchange and precipitation are a few mechanisms that result in immobilization and sorption. *Xanthomonas campestris*, *Streptococci* sp., *Pseudomonas aeruginosa*, *Pseudomonas oleovorans*, *Sphingomonas paucimobilis*, *Pasteurella multocida* and *Azotobacter vinelandii* are some of the recorded commercial rhizobacterial EPS strains that are capable of anionicity [256]. The association between positively charged metal ions and negatively charged EPSs and cell surfaces affects EPS-facilitated biosorption. In addition to causing or triggering biofilm formation in response to Cd contamination, *Herminiimonas arsenicoxydans*, a rhizobacterium with a Gram-negative phenotype, has also been reported to use EPSs to scavenge Cd when exposed to a 5 mM concentration [257]. This work demonstrates that, although rhizobacterial EPS production may not be increased in response to HM stress, produced EPS can still adsorb the metal.

Similarly, remediation of trace amounts of Pb and Cd has been studied using EPSs synthesized by *Marinobacter* sp. proteins and polysaccharides containing charged groups, such as carboxyl, amino, amide and hydroxyl groups, which often make up EPS. A synthesizing enzyme, Urease, first converts urea into ammonium ions in a microbially induced carbonate precipitation process. Generated ammonium ions then cause the pH to rise, decomposing the substrate urea into CO_3_^2−^, which triggers the precipitation of carbonates of HM ions or coprecipitation with CaCO_3_ [258]. The primary mechanism for Cd elimination is the synthesis of Cd carbonates induced by bacterial action. However, many safer rhizobacteria are dispersed in the environment and waiting to be uncovered for the remediation of HM-contaminated sites.

#### 6.2.3. Hydrogen Cyanide

**Figure 3:**
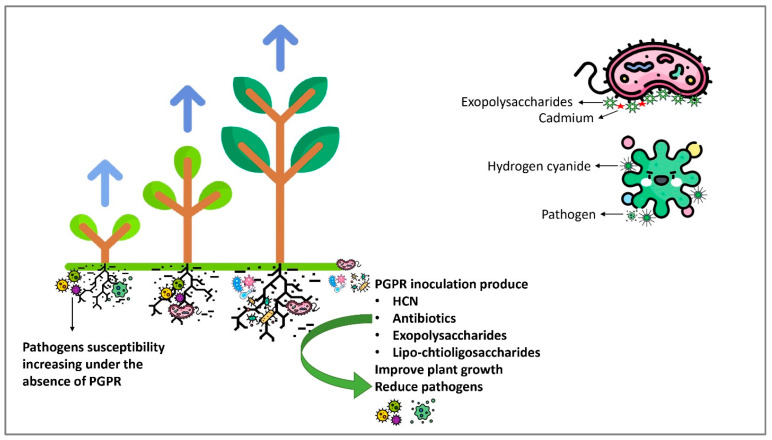
Microbially mediated indirect mechanisms for contaminant detoxification.

Rhizobacteria can produce volatile chemicals such as HCN, nitric oxide and hydrogen sulfide, as shown in Figure 3. Among these, HCN is a volatile substance that is crucial to the rhizosphere’s biology. According to Dimkić et al. [246], the biocontrol mechanisms of bacteria, such as those seen in certain *Pseudomonas* strains, often rely on secreted bioactive substances that target the pathogen, such as exoenzymes, antibiotics or HCN. Brahim and Ouhdouch [259] stated that phenazine-1-carboxamide, a phenazine formed by *P. chlororaphis* PCL1391, inhibited *F. oxysporum* from causing tomato root rot. Because of their speedy and vicious colonization of plant roots, fluorescent *Pseudomonas* has been deemed an effective biological control agent against soil-borne plant pathogens. They revealed that two processes were occurring: one included competition for nutrients in the rhizosphere, preferably at colonization sites, and the other involved the production of compounds such as siderophores, HCN and antibiotics. Rhizobacterial strains may produce HCN and affect the development of seedling roots in a range of plants [229]. In a collection of more than 2000 isolates, they found that 32% of bacteria were cyanogenic, with HCN levels ranging from trace to >30 nmol/mg cellular protein. *Pseudomonads* were the most vulnerable to cyanogenesis, facilitated by adding glycine to the culture medium. Previous studies have hypothesized that microbial HCN prevents pathogenic fungi from growing on their mycelia by preventing the production of ATP-mediated cytochrome oxidase. Microbial HCN promotes plant development and Cd mobilization in addition to biocontrol. The growth and Cd accumulation efficiency of *Sinapis alba* L. is significantly increased when the *Brevibacterium casei* MH8a strain produces HCN, ACCD and IAA [260]. The VOC produced by PGPR promotes plant growth and development, increasing shoot biomass and enhancing resistance to plants against Cd stress [261].

#### 6.2.4. Lipo-Chito-Oligosaccharides

Plants release flavonoids as secondary metabolites via their roots. It is well known that flavonoids have chemo-attractive properties that cause the expression of bacterial nod genes and the creation of lipo-chitooligosaccharides (LCO), which are crucial for developing nodules in roots. Flavonoids have selectivity for plant bacteria. Various chemicals may attract different bacterial species, making it feasible for a particular visitor to colonize. Because N fixation may encourage plant development in Cd-polluted soil, researchers are looking at N-fixing bacteria found in root nodules to find novel species that can be exploited in bioremediation. Rhizobia association with legume roots may improve Cd, Cu and Pb phytostabilization. Plants synthesize flavonoids to reduce Cd stress and increase antioxidant activity, and their release in the soil is a plant defense strategy. In particular, flavonoids may counter ROS within plant cells and can chelate metals such as Cd, Fe, Cu, Ni and Zn. The photo-microbiome community also affects the behaviors of each via by the methods of signal compounds [262]. Such types of signals are the holobiont’s hormones. For instance, riboflavin and lumichrome may act as microbes to plant signaling chemicals to promote plant development. Both substances have the potential to alter plant development significantly; lumichrome can hasten leaf emergence and leaf expansion. It may also enhance the plant’s height and total leaf area, further improving its biomass. Numerous plant species, including monocots and dicots, are affected by this [263]. It has been shown that microbe-to-plant signaling substances, including lipo-chito-oligosaccharides and thuricin, promote growth and development in various species of plants, especially under Cd stress [264]. Inoculating tomato seeds under Cd stress with a PGPR strain significantly boosted plant production of flavonoids and other phenolic compounds, accelerating antioxidant activity and reducing CD toxicity [265]. However, various safer LCOs, dispersed in the environment, are waiting to be uncovered for the remediation of Cd-contaminated sites.

## 7. Factors Affecting PGPR Bioremediation

The effectiveness of bioremediation is primarily influenced by site features, which can be further affected by environmental parameters such as water content, temperature, pH, nutrient availability, moisture content and pollutant bioavailability [266,267]. In addition, the bioremediation procedure is a complex system regulated and adjusted by multiple variables [30]. The bioavailability and biodegradation of Cd are influenced by interactions between contaminants, bacteria, the availability of nutrients and environmental variables [268]. The site location and its characteristics are the most significant factors impacting bioremediation. The degree and type of contaminants prevailing at the site determine the effectiveness of remediation [269]. These issues can be addressed through site assessment and prior inquiry before remediation.

Temperature plays a significant role in influencing the survival and growth of microorganisms [270]. By interfering with microbial metabolism, growth rate and the soil matrix, it plays a crucial part in microbe-assisted remediation by changing Cd physical and chemical states in polluted areas [271]. High temperatures, according to research by Javanbakht et al. [272], disrupt the metabolic activity of bacterial cells and impact the bioaccumulation process. Moreover, the physiological characteristics of microbes are influenced, which can speed up or slow down the remediation process. Temperature also affects how Cd ions interact with fungal membrane binding sites and influences the structure and stability of the fungal membrane by ionizing chemical moieties [273]. According to Jin et al. [185], *Simplicillium chinense* QD10 showed maximum biosorption efficiency for Cd and Pb at 30 °C, with 60.4 and 38.3%, respectively, which reduced significantly as temperature increased to 45 °C. Therefore, temperature regulation is critical for the success of the bioremediation process [274].

The metabolic activity of bacteria is influenced by pH, which can either accelerate or decelerate the elimination process. Bioremediation can be feasible over a wide pH range. However, according to Abatenh et al. [269], a pH range of 6.5 to 8.5 has the most significant potential for remediating most terrestrial and aquatic systems. The pH level affects the biosorption process by separating functional groups on fungal membranes and impacting the solubility and mobility of Cd [275]. The *Exiguobacterium* sp. exhibits a Cd biosorption capability that improves with an increased pH up to 7.0 and remains neutral when the pH is more significant than 7.0 [276]. pH and ionic strength can also impact microbial adsorption [274].

Similarly, nutrient availability, concentration and type are crucial for microbial activity and growth during bioremediation. Essential elements, such as carbon (C), nitrogen (N) and phosphorus (P), aid in the bacteria’s ability to manufacture the enzymes required to remediate Cd [21]. Lower nutrient availability impacts plants and microorganisms, eventually influencing bioremediation rate and efficiency. Adjusting the bacterial C:N:P ratio can increase the bioremediation efficacy by balancing essential nutrients such as N and P [274]. Providing adequate nutrients in an optimum environment promotes the metabolic activity of microorganisms, increasing the remediation rate [277,278]. According to reports, too much N in a contaminated medium leads to microbial inhibition [279].

Moreover, soil moisture levels may have adverse effects on microorganisms. Moisture affects the number and type of soluble materials and the pH and osmotic pressure of terrestrial and aquatic sites, affecting the efficiency of Cd remediation [274]. For bacteria to grow and metabolize efficiently, they typically require water activity levels between 0.9 and 1.0, with most bacteria thriving at the highest water activity values [280]. Therefore, the water content of polluted areas is a crucial variable that may influence the bioremediation rate. Recent research by Khodaverdiloo et al. [268] highlights that water scarcity, sodicity and salinity have recently been emphasized as significant factors affecting bioremediation effectiveness.

Unsuitable microorganisms or insufficient suitable microorganisms in contaminated locations also affect the bioremediation efficiency [270]. Because bioaccumulation is metabolically dependent and requires cellular energy for metal uptake, microbial biophysical processes also affect bioaccumulation. According to Srinath et al. [281], Vijayaraghavan and Yun [282] and Issazadeh et al. [283], it depends on different microbial traits, such as biochemical characteristics, genetic and physiological abilities, internal structure, cell surface qualities (including charge shifts) and surrounding environmental variables. Razmi et al. [22] discovered that various biological and chemical factors affect the effectiveness of phytoremediation. For plant-based remediation, root systems, which may have tap or fibrous roots depending on the depth of contaminants; aboveground biomass, which should not be preferred for livestock consumption; survival; and adaptations of plants, as well as plant growth, are essential considerations for choosing suitable plants [284]. Nonetheless, plant type has been identified as the primary determinant in Cd, Pb, Ni and Zn phytoremediation. Additionally, most fungal strains exhibit the highest biosorption efficiency under their ideal growing conditions [285].

The limited bioavailability of Cd in polluted soil has a significant impact on the effectiveness of bioremediation [7]. Several physicochemical processes, such as sorption, diffusion, desorption and dissolution, regulate the bioavailability of pollutants [12]. To manage this issue, several surfactants and chelating compounds increase Cd’s bioavailability for microbial breakdown and plant uptake [5]. Recent developments include the use of a variety of organic and inorganic chelating agents, including [S, S]-ethylenediamine succinic acid (EDDS), ethylenediamine tetra-acetic acid (EDTA), ethylenediamine-di-hydroxyphenyl acetic acid (EDDHA), nhydroxy-ethylenediaminetriacetic acid (HEDTA) and diethylene-triaminepentaacetic acid (DTPA). According to Sarwar et al. [286], these chelating agents have successfully demonstrated their ability to form a complex with HMs and boost bioavailability.

## 8. Recent Advancements (Genetic and Metabolic Engineering), (Membrane and Enzyme Technology), (Metagenomics Approaches) and (Nanoparticle Technology)

### 8.1. Membrane and Enzyme Technology 

The remediation of HMs, i.e., Cd, using microbial enzymes, is significantly more efficient than other bio-remediation techniques, as they are ecologically friendly, affordable and innovative [287]. Research is being conducted on the ability and affordability of enzymes, including oxidoreductases, nitro-reductases and dehalogenases, to detoxify the harmful effects of Cd in agricultural soils [288]. Exudates secreted by plants are used as carbon and energy sources by PGPR in conjunction with plant roots to produce the enzyme 1-aminocyclopropane-1-carboxylase deaminase and IAA, which are used to break down metal pollutants [289,290]. These enzymes must be utilized under ideal temperatures and pH conditions to degrade pollutants effectively. The harmful effect of bioactive metals such as Cd can be alleviated using physicochemical characteristics due to different enzymes [291]. The development of the enzyme phytochelatin synthase and the production of proline can quickly bind to HMs at toxic concentrations and act as a free radical scavenger, respectively [292,293]. Phytochelatins (PCs) are thiol-rich and short-chain repetitions of low-molecular-weight peptides produced by phytochelatin synthase from glutathione to activate plant defense against metal stress [294]. 

Microbially induced precipitation (MIP) is a critical aspect of the biogeochemical cycle, wherein ions or chemicals react with metabolic products released by microbes, leading to the deposition of mineral particles [294]. One of the crucial features is microbially induced carbonate precipitation (MICP), which reduces Cd’s mobility by utilizing a carbonate-biomineralization microbe’s metabolic activity. Microorganisms release urease, which produces carbonate by reacting with urea [295]. To build Cd carbonate crystals, first, Cd ions are coupled with cell binding sites such as carboxyl, phosphate, cyanide, imidazole, and amino binding sites [296,297] and then are catalyzed by carbonate to form Cd carbonate (CdCO_3_) crystals via bacterial urea hydrolysis [298]. Cd carbonate reaches a saturation state in solution due to its poor solubility. Burns et al. [299] showed that functional groups on a microbe’s surface mediate microbial cell adhesion to mineral surfaces, and Huang et al. [300] found that microbial discharge mediates the deposition of mineral particles.

Consequently, the removal percentage outperformed individual biosorption. Therefore, MICP based on biomineralization is an ideal method for removing Cd contamination at 10–50 mg L^−1^. A promising method for the microbial remediation of metals is oxidation–reduction, in which enzyme reactions produce fewer toxic species by altering the valence state of polyvalent metal ions [301]. The activity of soil enzymes is sensitive to many forms of HM contamination, as HM pollution can reduce the activities of different enzyme-contaminated soil [302]. Their activity can be upregulated by following a proper enzyme-based remediation process [303,304]. However, significant research is needed to develop innovative methods that might be more precise and effective than those that are currently available [305]. Moreover, a significant gap exists between laboratory-level study, bioreactor/scaling-up applications and field research.

### 8.2. Genetic and Metabolic Engineering 

Microorganisms can remediate HMs such as Cd by metabolizing them into innocuous by-products through co-metabolism [306]. Making new pathways, altering existing gene sequences and introducing single genes or operons into microorganisms are the most frequently utilized approaches [306]. Enzymatic bioremediation is a straightforward, efficient and eco-friendly method for microbe-assisted removal and destruction of persistent xenobiotics [307]. Genetic engineering for bioremediation seeks to alter plants, microbes and enzymes to make them viable tools for breaking down hazardous materials [308]. The bioremediation of Cd can be facilitated via these approaches [309]. Applying genetically modified bacteria and plants during the bioremediation of Cd-contaminated soils and other organic pollutants has become a promising technique [310]. Most methods include locating and inserting metal-uptake-related genes into plants and competent bacterial cells. n-alkane-degrading microbes possess specific genes, including xylE, polycyclic aromatic hydrocarbons (alkB, alkB1, alkB2 and alkM) and aromatic hydrocarbons (ndoB and nidA), which are used as markers to identify microbial-assisted biodegradations. Metabolic engineering emphasizes microbial-based enzymes involved in various degradation processes, such as oxidases, esterases, phenoloxidases, monooxygenases and oxidoreductases [311]. Several enzymes, including mixed-function oxidases (MFO), laccase, glutathione-S-transferase and cytochrome P-450, participate in the biodegradation of contaminants [312]. Enzyme immobilization also considerably increases enzyme activity, half-life and stability [307]. The stability of microbes must be maintained before their field application to use GEMs for bioremediation because the stability of recombinant plasmids inserted into the organism is responsible primarily for the catabolic activity of released GEM [313]. According to Dixit et al. [88], biosensors can estimate concentrations of HMs such as Cd in contaminated areas. However, variations in reaction times, detection thresholds, sensitivity, signal relaxation lengths and stability can limit the application of biosensors [314]. Modern genetics and omics techniques have made it possible to study the catabolism of contaminants using different microorganisms, allowing scientists to better understand the ecology, physiology and biochemistry of microorganisms that remediate Cd [313]. The drawback of alternative culture-independent techniques has been overcome by DNA microarrays, a high-throughput method that can identify several genes in a single test. The most popular gene array method for examining gene function is the GeoChip array [314]. Recombinant DNA technology is crucial for bioremediation because it helps to analyze, monitor and evaluate the specific technique. Nevertheless, it must be used responsibly and follow biosafety guidelines.

### 8.3. Metagenomics Approaches

Metagenomics is a rapidly expanding and new field of study. It is an environment-friendly and practicable technique for analyzing genetic material extracted directly from environmental samples. Therefore, in a niche ecosystem, metagenomics provides information through sequence and function-based research techniques about the communities of microbes of non-cultivable organisms. It comprises methods such as shotgun metagenomics, high-throughput sequencing and bioinformatics that aid in identifying, characterizing and screening prospective species [315,316]. Metagenomics is important for detecting and monitoring microbial activities [317]. Using metagenomics, DNA can be studied from ambient samples without isolating and cultivating the microbes. This method was first applied to find new microbes and microbial products and to sample the microbial diversity from various environmental niches to analyze their ability to eliminate organic pollutants [318]. Metagenomics is a fast-growing and emerging area of study. Opportunities to discover new ecosystems are made possible by the function-focused metagenomic technique, which promotes the discovery of new genes and provides genetic analysis [319]. Sequence interpretation, regarded as essential for feature estimates, is the foundation of the sequence-based technique. The bioremediation technique also considers microbial diversity and particular genes identified by metagenomic research with the potential to act as pollution biomarkers with bioactive substances and enzymes determined using metagenomics techniques. Additionally, metagenomics approaches are useful in identifying the enzymes, metabolites and bioactive substances produced by bacteria, all of which play a vital role in water treatment. Metagenomics studies analyze strategies to uncover new genes and bacteria to mediate the detoxification of organic pollutants, including Cd [320,321].

In Cd-contaminated soils, the relative abundance of Kyoto Encyclopedia of Genes and Genomes (KEGG) pathways increased with an enriched metabolism, biosynthesis and the degradation of different fatty acids and nucleotides, which was connected to microorganisms’ induced tolerance of Cd [316]. In addition to investigating the studied soil microbial population, their roles and genes for diverse applications, soil metagenomics can be envisioned as a technique for promoting plant growth in Cd-contaminated soils [322]. Metagenomic sequencing depends upon several strategies that may explore new ways to identify bioremediation contaminants. Pyrosequencing, single-molecule sequencing, ligation sequencing and reverse terminator sequencing are examples of next-generation sequencing techniques made possible by technical drift and enable high-throughput readings in a shorter time. Metagenomics is one of the best breakthroughs now available, and it has frequently been used in building better ecosystems through bioremediation. The Simple Metagenomics Analysis Shell for the community of microbes is the only pipeline based on metagenomic investigations to share the ideas and events of architecture with Smash-Cell [323]. This allows comparing multi-metagenome compositions, making functional graphical representations of these tests and estimating metagenome quantitative phylogenetic and functional compositions. The most common method is Meta Genome Analyzer (MEGAN), which uses sequence data for a practical analysis across various bioinformatics applications [324]. This approach is perfect for evaluating metagenomics and metaproteomics using interactive functional and taxonomical data. A sophisticated metagenome tool called the Metagenome Subsystems Technology Rapid Annotation (MGRAST) provides microbial communities with quantitative insights based on sequencing data [325]. The Integrated Microbial Genomes and Metagenomes (IMG/M) system supported the creation of genome and metagenome databases of different microbes. 

### 8.4. Nanoparticle Technology

Nanotechnology has emerged as an attractive field to synthesize and modify innovative and nanostructured materials for several purposes, including the remediation of the environment from organic and inorganic pollutants, known as nano bioremediation. The removal of Cd pollution could be more effective with the available technology. However, novel physical and chemical characteristics of nanoparticles (NPs) can therefore be used to remediate Cd efficiently [326]. With their unique chemical, structural and multifunctional features, morphologies, various compositions and high mechanical strength, NP-based innovative materials and nanochemistry methods are promising for degradation, adsorption and catalysis applications in metal pollution [258,327,328]. Various bulk materials can be used to create NPs, and particles’ size, shape and chemical makeup all influence the behavior and composition of NPs [329]. They have paved the way for low-cost and effective ways to limit the toxic effects of environmental contaminants [330]. Nanotechnology usage has become a fiercely debated topic due to the need for more thorough investigations and knowledge of how NPs interact with other environmental elements. Silver NPs impact the microbial populations in the root zone, aiding in the removal of Cd [331]. The use of NPs improves the phytoremediation of HMs, including Cd, by upregulating the growth of plant roots and shoots [332]. Engineered nanoparticles (ENPs), a new class of environmental chemicals, have shown considerable effects on the fate and transport of coexisting ecological contaminants and the effectiveness of plant uptake [333].

Most studies investigating the effects of ENPs on plant metal uptake have focused on metallic ENPs. This makes sense, given that some metallic ENPs tend to dissolve in the root zone, leading to dissolved ions that may compete with metal ions for plant uptake, altering the course of HM uptake [334]. For example, the uptake of Cd was significantly reduced by the application of citrate-coated magnetic NPs in wheat, which ameliorated Cd toxicity. In addition, the uptake of Cd was reduced considerably by the application of TiO_2_NPs in rice [335]. The authors also examined how cerium oxide (CeO_2_) NPs affected the amount of co-occurring Cd that accumulated in soybeans (*Glycine max*) and found that CeO_2_NPs considerably altered the amount of Cd that accumulated, hindering its concentration in soybean shoots [336]. However, there is still limited research on how ENPs affect the uptake of coexisting Cd by plants. More mechanical knowledge about these interactions in the plant rhizosphere must be determined. Further research is necessary to better understand the complex chemical and biological processes that may affect plant Cd uptake in the presence of ENPs.

## 9. Challenges and Future Prospects

Various bioremediation techniques have been successfully in restoring contaminated locations exposed to Cd. However, when using bioremediation techniques, there are a few crucial considerations to remember. Before suggesting bioremediation, there is a requirement for the regular study and assessment of the amount of Cd and other pollutant concentrations in polluted locations. Choosing suitable microorganisms and plant species is difficult for sites with several metals such as Cd and other organic contaminants. Second, hazardous metals and metalloids such as Si, Hg and As at the site may volatilize into the atmosphere during plant-based bioremediation, posing a risk to living things. Third, edible plants used for bioremediation could be eaten by animals or insects, which could further contaminate the food chain and eventually reach people, posing a significant health concern. Therefore, phytoremediator plants that are neither edible nor palatable should be preferred. Sufficient precautions must be taken during the cultivation and harvesting of edible plants to prevent further issues. In situ, phytoremediation is more challenging when Cd is present more deeply in the soil, where plant roots cannot reach.

More research, assessments and inquiries are needed to improve our knowledge and comprehension of optimum management techniques for the practical bioremediation of Cd. Mechanisms, metabolites and new approaches/methods need to be clarified in a futuristic manner. Using hyperaccumulator plants to remove Cd from polluted soil effectively requires unique techniques for the continued development of plant-based bioremediation to be easy and effective. This can be performed in two different ways: first, by discovering and validating new species of diverse hyperaccumulator plants, and second, by creating hyperaccumulator plants through genetic engineering. Moreover, we can consider deep-rooted hyperaccumulator plants, such as woody plants or trees such as *Populus canescens*, *Schima superba*, *Rinorea bengalensis* and *Pycnandra acuminata*, which have higher growth rates, biomass and translocation rates, as well as more tolerant plant species.

Biotechnological interventions, such as genetic engineering, can increase a known metabolic pathway’s transfer and biodegradation rates and enhance the accumulation of Cd or the degradation of recalcitrant compounds by introducing a completely new metabolic pathway into the microbe. Moreover, it may be possible to remediate Cd from the soil by overexpressing foreign genes into a non-tolerant plant with a larger biomass. Modifying microbial niches that increase resistance against Cd pollution will be made easier with the help of the cutting-edge method of the holo-genomics analysis of plant microorganisms. There is a need to design suitable amendments for multi-metal-contaminated and multi-stress environmental situations to improve the survival of suitable plant species. Even though several organic and inorganic amendments and metal chelators are available, additional research is required to identify more effective and environmentally acceptable amendments that may be used to treat soil exposed to several metal contaminants and stresses. The effectiveness and dependability of bioremediation depend on the collaboration and contribution of researchers, scientists, policymakers, governments, industrial sectors and individuals.

## 10. Conclusions

High levels of Cd are being released into the environment due to human activity, directly and indirectly affecting all living things. Reports have indicated that contaminated soil contains numerous HMs simultaneously, and conventional detoxification techniques are less effective than the bioremediation procedure. It has been established that bioremediation procedures are significantly cheaper than other physicochemical remediation methods. Numerous bacterial and fungal strains have recently been isolated and characterized from metal-contaminated and mining-abandoned soils. Many strains of *Bacillus* spp., *Pseudomonas* spp., *Aspergillus* spp. and *Penicillium* spp. are present and exhibit excellent metal resistance in soil, especially against Cd. A variety of contaminated areas throughout the world are currently using bioremediation, with variable degrees of effectiveness. The main worry for the considerable yield of bioremediation is the inclusion of appropriate supplements and improving environmental conditions. To address the issue, adding organic matter and a group of microorganisms can increase microbial metabolic activity and enhance the potential for bioremediation. Moreover, more research is still needed to identify the best microbes and hyperaccumulator plants with a high tolerance for multi-metal-contaminated and multi-stress environmental locations and to accumulate several metals simultaneously. More emphasis will be paid to plant–microbe-based bioremediation strategies to find novel plant–microbe pairs with high metal removal effectiveness and to create an environment that is conducive to other microbial strains. This will indirectly improve the health of the soil. Additionally, more research is required on combining nanomaterials, biochar and microorganisms with bioremediation.

## Figures and Tables

**Table 1:** Impact of Cd toxicity on different plant species.

Plant Species	Level of Cd	Changes/Damages	References
**Seed germination and seedling growth**			
*Pisum sativum* L.	20–500 µM	Inhibition of proteolytic enzymes and restriction of starch metabolism, leading to the failure of protein mobilization in seeds.	[73]
*Ocimum basilicum* L.	20 mg kg^−1^	Alterations in the embryo and reductions in the oil contents of seeds.	[74]
*Brassica oleracea* L.	5 mg L^−1^	Decreased seed germination with an increase in MDA contents, electrolyte leakage and H_2_O_2_ contents.	[75]
*Sassafras tzumu* Hemsl.	100 mg kg^−1^	Restricted seedling growth and germination, and impairment of photosynthesis at higher doses.	[63]
*Brassica juncea* L.	15 mg kg^−1^	Disintegration occured in roots and shoots, and levels of ROS increased in plant shoots.	[76]
*Oryza sativa* L.	50 μM	Lower seed germination rate due to the hyperaccumulation of Cd.	[53]
*Zea mays L.*	100 mg kg^−1^	Reduced seedling growth and activity of cellular antioxidants.	[77]
**Growth and development**			
*Cicer arietinum* L.	50 μM	Reduction in growth and appearance of symptoms of necrosis and chlorosis in leaves.	[78]
*Ipomoea aquatica* Forsk		Reduced growth and development of root and shoots.	[79]
*Lens culinaris*	50 µg g^−1^	Increased electrolyte leakage and ROS production, resulting in lower plant growth.	[80]
*Medicago sativa* L.	10 mg kg^−1^	Higher concentrations damaged proteins, changed cell wall infrastructure and metabolism, and limited growth.	[81]
**Osmolytes and photosynthesis**			
*Cajanus cajan* L.	10 mg kg^−1^	Lower organic osmolytes ultimately caused a disturbance in osmotic adjustments.	[82]
*Vigna angularis*	64 mg L^−1^	Cellular antioxidants decreased at higher concentrations, resulting in the lower production of low-molecular-weight osmolytes.	[83]
*Zea mays* L.	150 μM	Reduction in photosynthetic pigments and gas exchange traits.	[58]
*Coriandrum sativum* L.	20 µM L^−1^	Inhibited gas exchange traits and biochemical processes.	[45]
*Capsicum annuum* L.	500 ppm	Induction of stomatal closure, resulting in decreased photosynthetic pigments, a smaller stomatal size and reduced transpiration.	[84]
*Mentha arvensis*	150 mg Kg^−1^	Reductions in mineral assimilation, photosynthetic attributes and photosynthetic pigments occurred.	[85]

**Table 2:** Microbe-assisted phytoremediation of cadmium from soil under different experimental settings.

Experiment	Contamination Level	Microorganisms	Plant	Results	References
Pot	10 mg kg^−1^	*Pseudomonas fluorescens*	*Hordeum vulgare*	Phyto-stabilization of Cd due to PGPR activity, increased uptake of essential plant nutrients and enhanced plant growth attributes.	[90]
Greenhouse pot	10.7 mg kg^−1^ Cd	*Bacillus* spp.	*Solanum nigrum*	Increased plant growth attributes under Cd stress, enhanced absorption of P and Fe as well as increased Cd contents in aerial plant parts.	[91]
Incubation study	200 μg/mL	*Klebsiella michiganensis*	*Oryza sativa*	Cd bioaccumulation by tolerant bacteria with a concurrent decline in its uptake by plants.	[92]
Pot	50 mg kg^−1^	*Cupriavidus necator*, *Sphingomonas* and *Curtobacterium* spp.	*Brassica napus*	Increased plant biomass and growth traits under Cd contamination in inoculated treatments along with enhanced Cd uptake by aerial plant parts.	[93]
Pot	0, 50, and 100 mg L^−1^	*Rhizobium pusense*	*Glycine max*	Decreased soybean root Cd contents by 45.9 and 35.3%, respectively, at contamination levels of 50 and 100 mg L^−1^.	[94]
Pot	(0, 25, 50, 75, 100, 150 and 200 mg kg^−1^)	*Enterobacter cloacae*, *Klebsiella pneumonia* and *Klebsiella* spp.	*Pennisetum giganteum*	Combined application of rhizobacteria increased the bioaccumulation factor of Cd for plants.	[95]
Pot	0, 5, 10, 15 and 20 mg kg^−1^	*Serratia marcescens*	*Chrysopogon zizanioides*	Increased phytoaccumulation of Cd, soil biological health, as well as antioxidative potential of plants under bacterial inoculation. Maximum Cd phytoextraction in roots (289.47 mg kg^−1^), leaves (59.38 mg kg^−1^) and stem (88.33 mg kg^−1^) with a concomitant increase in plant biomass (9.68–45.99%).	[96]
Field	2.2 mg kg^−1^	*Rhizobium leguminosarum*, *Bacillus simplex*, *Luteibacter* sp. + *Variovorax* sp., *Pseudomonas fluorescens*	*Lathyrus sativus*	Increased growth attributes as well as nodule number, and plant nutrient uptake, and phytoaccumulation along with reduced rhizosphere concentration of Cd (61%).	[97]
Pot	50 and 100 mg kg^−1^	Fungi “*Funneliformis mosseae*” and bacteria *Enterobacter* sp. and *Enterobacter ludwigii*	*Lycopersicon esculentum*	Increased dry weights of shoots (119–154%) and roots (91–173%) under combined inoculation. Furthermore, decreased Cd concentrations in shoots as well as translocation factors under inoculated treatments were observed.	[98]
Pot	0, 0.25, 0.5, 0.75 and 1 M CdSO_4_	*Serratia marcescens*	*Oryza sativa*	Increased Cd removal from soil (66 mg kg^−1^ after 20 days).	[99]
Incubation study	0, 0.25, and 0.5 mM Cd	*Stenotrophomonas maltophilia*	*Capsicum annuum*	Under Cd stress, increased root lengths (1.46 times) in the inoculated treatment compared to the control.	[100]
Pot	15 mg kg^−1^	*Variovorax paradoxus*, *Rhizobium leguminosarum* and fungus *Glomus* spp.	*Pisum sativum* and *Brassica juncea*	More prominent positive effect of consortium inoculation on *Pisum sativum* rather than *Brassica juncea*, in terms of growth, nutrient uptake and increased seed Cd concentration.	[101]
Greenhouse pot	2.12 mg kg^−1^	*Bacillus megaterium*, *Glomus mosseae*, and *Piriformospora indica*	*Solanum nigrum*	Cd accumulation (104%) observed under the combined application of *Bacillus megaterium* and *Glomus mosseae* in addition to increased soil biological health under contaminated conditions.	[102]
Pot	100 mg kg^−1^	*Bacillus* sp.	*Oryza sativa*	Reduced bioavailable Cd concentration (39.3%), increased phytoextraction efficiency of rice for Cd (48.2%) and increased rice growth and yield traits under inoculation compared to the control.	[103]
Greenhouse	0.064 mg L^−1^	*Klebsiella huaxiensis* and *Pantoea cypripedii*	*Pennisetum purpurenum*	Enhanced Cd phytoaccumulation in all variants of the tested plant (28.43–38.07 mg kg^−1^).	[104]
Pot	30 μmol L^−1^	*Enterobacter cloacae*	*Solanum nigrum*	Increased soil Cd phytoextraction by plants along with increased plant growth under Cd stress.	[105]
Pot	0, 0.25, and 0.50 mg kg^−1^	*Bacillus* spp.	*Oryza sativa*	Increased Cd immobilization in soil by its surface adsorption concomitant with increased plant growth.	[106]
Incubation	0.4 mM CdCl_2_	*Pseudomonas aeruginosa* and *Burkholderia gladioli*	*Solanum lycopersicum*	Alleviation of Cd toxicity in plants was evident by an increase in phenolic compounds, osmolytes and low-molecular-weight organic acids.	[107]
Growth room trial	0–400 μg/mL	*Enterobacter cloacae*	*Oryza sativa*	Increased Cd removal efficiency (72.11%) against a contamination level of 400 μg/mL	[108]
Pot	2 g	*Curtobacterium oceanosedimentum*	*Capsicum frutescens*	Increased root (58%) and shoot (60%) lengths, enhanced accumulation of Cd in roots compared to shoots under bacterial inoculation.	[109]
Pot	1.68 mg kg^−1^	*Buttiauxella*, *Pedobacter*, *Aeromonas eucrenophila*, *and Ralstonia pickettii*	*Sedum plumbizincicola*	Inoculation led to reduced reducible and residual Cd and increased Cd availability coefficients by 1.15–6.41 units. Cd contents in shoots (29.63–46.01%) and roots (11.42–84.47%), bioconcentration factor (2.13–2.72) and Cd removal rate (48.25%) compared to the control treatment	[110]

**Table 3:** Microbial biosorption by different microbes.

Species name	Initial Cd Concentration	Experimental Medium	Cd Remediation Efficiency	References
**Bacterial species**				
*Pseudomonas fluorescens* and *Bacillus subtilis*	150 mg L^−1^	Soil	16.7	[162]
*Pseudomonas sp. DDT-1*	0.9 mg kg^−1^	Soil	40.3	[163]
*Kocuria rhizophila*	150 mg L^−1^	Aqueous	9.07 mg g^−1^	[164]
*Klebsiella michiganensis*	1000 µg ml^−1^	Soil	97%	[165]
*Enterobacter* sp	3500 µg ml^−1^	Soil	95%	[166]
*Rhodobacter sphaeroides*	65.33 mg kg^−1^	Soil	30.7	[167]
*Bacillus aryabhattai* and *Bacillus amyloliquefaciens*	250 mg L^−1^	Soil	96%	[168]
*Aspergillus sydowii*	50 mg kg^−1^	Soil	10.44%	[169]
*Cupriavidus* sp.	13.82 mg kg^−1^	Soil	58.2%	[170]
*Paenibacillus* sp. and *Bacillus* sp.	20 mg L^−1^	Soil	128.50%	[171]
*Bacillus* sp. TZ5	150 mg L^−1^	Soil	48.49%	[172]
*Acidithiobacillus caldus* DX, *Acidithiobacillus thiooxidans* DX, *Acidithiobacillus thiooxidans* ZJ, *Acidithiobacillus thiooxidans* AO1, *Ferroplasma acidiphilum* DX, *Acidithiobacillus caldus* S1 and *Leptospirillum ferriphilum* DX	9.09 mg kg^−1^	Soil	32.09%	[173]
*Cupriavidus* sp. (KU168590), *Ensifer* sp. (KU168586), *Burkholderia* sp. (KU168588), and *Paenibacillus* sp. (KU168587)	0.21 mg kg^−1^	Soil	33.0%	[174]
*Enterobacter cloacae*, *Pseudomonas aeruginosa*, and *Klebsiella* *Edwardsii*	50 mg L^−1^	Soil	58.80%	[175]
*Bacillus subtilis*	147.75 mg kg^−1^	Soil	35.17%	[176]
*Burkholderia* sp. and *Bacillus* sp.	5 mM	Soil	84.17%	[177]
*Bacillus* sp.	49 mg kg^−1^	Soil	43.53%	[178]
*Bacillus subtilis*	147.75 mg kg^−1^	Soil	18.56%	[179]
*Firmicutes* sp. and *Proteobacteria* sp.	14.9 mg kg^−1^	Soil	40.0	[180]
*Enterobacter hormaechei* SFC3	100 µg ml^−1^	Soil	90.21%	[181]
*Streptomyces pactum* Act12 and *Streptomyces Roche* D74	1.62 mg kg^−1^	Soil	56.39%	[182]
*Bacillus velezensis*	-	Soil	1.65 μg g^−1^	[183]
**Fungi**				
*Aspergillus niger*, *Aspergillus fumigatus*, and *Penicillium rubens*	0.6 mg L^−1^	Soil	79%	[184]
*Simplicillium chinense*	400 mg L^−1^	Soil	88%	[185]
*Aspergillus flflavus*, *Aspergillus gracilis*, *Aspergillus penicillioides*, *Aspergillus restrictus*, and *Sterigmatomyces halophilus*	1000 mg L^−1^	Soil	95%	[186]
*Phanerochaete chrysosporium*	25 mg L^−1^	Soil	96%	[187]
*Agaricus bisporus*, *Pleurotus platypus*, and *Calocybe indica*	-	-	98.97	[188]
*Lactarius piperatus and Agaricus bisporus*	265 mg L^−1^	Aqueous	95%	[189]
*Rhizophagus intraradices*	-	Soil	38%	[190]
*Trichoderma harzianum*	147.75 mg kg^−1^	Soil	47.69%	[176]
**Algae/cyanobacteria**				
*Asparagopsis armata*	150 mg L^−1^	Aqueous	10.6%	[191]
*Chaetoceros calcitrans* and *Tetracelmis chuii*	-	Aqueous	-	[192]
*Ulva lactuca*	80 mg L^−1^	Aqueous	41.0%	[193]
*Chara aculeolate*	-	Aqueous	23 mg g^−1^	[194]
*Chlorella pyrenoidosa*	1.5 mg L^−1^	Aqueous	45.45	[195]
*Scenedesmus acutus*	1.5 mg L^−1^	Aqueous	57.14	[195]

## Data Availability

Not applicable.
